# Association Analysis of the *FTO* Gene Polymorphisms with Growth and Carcass Traits of Heying Black Chicken and Tissue Expression Profile

**DOI:** 10.3390/ani15182718

**Published:** 2025-09-17

**Authors:** Hao Ding, Lan Chen, Can Chen, Tao Zhang, Weilin Chen, Genxi Zhang, Jinyu Wang, Kaizhou Xie

**Affiliations:** 1 College of Veterinary Medicine, Yangzhou University, Yangzhou 225009, China; chenlan9326@163.com (L.C.); chencan19981224@163.com (C.C.); 2 Joint International Research Laboratory of Agriculture and Agri-Product Safety, Ministry of Education, Yangzhou University, Yangzhou 225000, China; a934263381@163.com (W.C.); gxzhang@yzu.edu.cn (G.Z.); jywang@yzu.edu.cn (J.W.); kzxie@yzu.edu.cn (K.X.); 3 College of Animal Science and Technology, Yangzhou University, Yangzhou 225009, China

**Keywords:** *FTO*, carcass traits, growth traits, Heying black chicken

## Abstract

Fat volume and obesity-related genes (e.g., the *FTO* gene) are important candidate genes affecting energy metabolism. Single nucleotide polymorphisms (SNPs) in the *FTO* gene are associated with carcass, growth and meat quality traits of pigs, cattle, sheep, rabbits and ducks. The purpose of this study was to detect the single nucleotide polymorphisms in the chicken *FTO* gene coding region by DNA sequencing and analyze its association with the carcass and growth traits of Heying black chickens. We detected polymorphisms in exons 5, 7, 8 and 9, respectively, g.57337C>A, g.64757T>G, g.97213G>A and g.220985G>A, which are synonymous mutations. g.57337C>A mutation site CA and AA genotype individuals were significantly higher than *CC* genotype individuals in live weights, head weights, breast muscle weights and leg muscle weights (*p* < 0.05), *AA* genotype individuals were significantly higher than *CC* genotype individuals in slaughter live weights and liver weights (*p* < 0.05) and *CA* and *AA* genotype individuals were significantly higher than *CC* genotype individuals in heart weights (*p* < 0.01). In terms of growth traits, the weights of individuals with genotype *CA* at 8 weeks were significantly higher than that of individuals with genotype *CC* (*p* < 0.05), and the weights of individuals with genotype *AA* and *CA* at 10 weeks and 16 weeks were significantly higher than that of individuals with genotype *CC* (*p* < 0.05). For the g.64757T > G mutation, individuals with the TT genotype exhibited significantly higher values (*p* < 0.05) than those with the TG genotype across multiple traits, including slaughter weight, live weight, eviscerated weight and semi-eviscerated weight. Individuals with *GG* genotypes were significantly higher than individuals with *TG* genotypes (*p* < 0.05) in slaughter weights and wing weights. In terms of growth traits, the 16-week-old body weight of individuals with *TT* genotype was significantly higher than that of individuals with *TG* genotype (*p* < 0.01). The *GA* genotype exhibited significantly higher slaughter weights in the g.97213G>A variant compared to the *GG* genotype (*p* < 0.01), and in live weights, eviscerated weights, semi-eviscerated weights, leg muscle weights and wing weights, *GA* genotype was significantly higher than in *GG* genotype (*p* < 0.05). In terms of growth traits, *GA* genotype was significantly higher in individuals 8 weeks old, 10 weeks old and 16 weeks old than *GG* genotype (*p* < 0.05). g.220985G>A was significantly higher in individuals with *GG* genotype than *GA* genotype (*p* < 0.05). In terms of growth traits, the weight of *GG* genotype was significantly higher than that of *GA* genotype (*p* < 0.05). The results showed that the *FTO* gene may be a candidate gene related to chicken growth and slaughter traits and lays a foundation for Heying black chicken assisted breeding.

## Simple Summary

Analyzing the genetic basis of important economic traits of livestock and poultry and screening relevant molecular markers are the theoretical basis and premise of using molecular breeding technology to improve relevant traits. The *FTO* gene, which regulates adipogenesis and fat accumulation and is strongly associated with body fat index, was the first disease-associated gene to be identified using gene-wide association analysis (GWAS) in humans. However, the association between the *FTO* gene polymorphism and important traits of chicken is still poorly understood. It is not clear whether SNPs in the *FTO* gene can be used as markers for the molecular marker-assisted breeding of important economic traits in chickens. In the present study, SNPs in exons of the *FTO* gene were detected by PCR amplification and DNA sequencing.

## 1. Introduction

The long-term goal of broiler genetic improvement is to enhance meat production performance and growth traits. In the 21st century, molecular breeding technology represented by functional genomics, molecular marker-assisted breeding and gene editing has become an important symbol and development direction of animal breeding. Analyzing the genetic basis of important economic traits of livestock and poultry and screening relevant molecular markers are the theoretical basis and premise of using molecular breeding technology to improve relevant traits. Single nucleotide polymorphism (SNP) was first proposed in 1997 [1]. As a third-generation genetic marker, SNPs have been widely used in population genetics and disease-related gene localization research, and play an important role in early diagnosis, prevention and treatment of diseases, study of the influence of genetic factors in drug metabolism and guidance for drug clinical use [2]. With the rapid development of molecular biology and cross-cutting disciplines, the variety of SNP detection techniques is increasing. In addition to the common sequencing, allele-specific amplification and high-resolution melting curve methods, there are also mass spectrometry, gene chips, oligonucleotide linkage analysis, dynamic allele-specific hybridization, and endonuclease-based restriction fragment length polymorphisms and random amplification polymorphisms [3]. SNP detection will become more efficient and rapid, and SNP-based research will become increasingly widespread, whether in molecular marker-assisted breeding, rapid identification of specific strains or even between individuals, research into resistance to disease in animal organisms or individualized drug delivery.

With the rapid development of molecular biology technology, SNP gene detection and typing technology and methods continue to innovate. Although some classical SNP detection technologies are still widely used in research, highly sensitive and high-throughput SNP detection methods are also increasingly valued. For example, the most widely used SNP detection method in research is PCR-DNA direct sequencing, namely DNA Sanger sequencing [4]. Ye used SNP arrays to genotype 450 roosters [5]. Zhang directly sequenced chickens and detected AMY1A polymorphisms in chickens. Seven SNPs in the 5′ flanking region, exon, intron, and 3′ UTR (3′ untranslated region) of *AMY1A* were significantly associated with daily weight gain, average daily feed intake, leg muscle weight and abdominal fat [6].

The Fat mass and obesity-associated (*FTO*) gene is an m6A demethylase belonging to the ALKB dioxygenase family [7]. The FTO protein is an AlkB-like DNA/RNA demethylase with a strong preference for 3-methylthymidine (3-meT) in single-stranded DNA or 3-methyluracil (3-meU) in single-stranded RNA [8,9,10]. The catalytic activity of the *FTO* gene is generated through the interaction of the amino-terminal and carboxy-terminal structural domains. The *FTO* gene possesses an additional loop that selectively competes with the unmethylated strand of the DNA double-stranded to bind the *FTO* gene, suggesting that it has an important role in the selection of duplex nucleic acids by the *FTO* gene [11]. The *FTO* gene, which regulates adipogenesis and fat accumulation and is strongly associated with body fat, was the first disease-associated gene to be identified using gene-wide association analysis (GWAS) [12]. The *FTO* gene has also been associated with type 2 diabetes [13], growth retardation, developmental delay [14], metabolic disorders [15] and hypertension [16]. In addition, the *FTO* gene also plays a crucial role in muscle growth, as it can promote the development of bovine muscle satellite cells, which are mainly responsible for muscle growth and regeneration [17].

The polymorphisms of the *FTO* gene and their associations with economic traits have been studied in pigs [18], cattle [19], sheep [20], rabbits [21] and ducks [22]. However, the association between the *FTO* gene polymorphism and important traits of chicken is still poorly understood. Muscle development is intricately linked to an animal’s growth rate, meat yield and quality, while also serving as an indicator of its health status and feed utilization efficiency. Slaughter characteristics, on the other hand, dictate the economic value, quality and safety of meat products, reflect breeding management standards and cater to market demands and consumer preferences. Both factors are pivotal in enhancing the efficiency of animal husbandry. It is not clear whether SNPs in the *FTO* gene can be used as markers for the molecular marker-assisted breeding of important economic traits in chickens. In the present study, SNPs in exons of the *FTO* gene were detected by PCR amplification and DNA sequencing. The association between the *FTO* gene polymorphism and carcass traits and growth traits of Heying black chicken was analyzed. We hope to provide a theoretical basis for the molecular marker-assisted breeding of Heying black chicken.

## 2. Materials and Methods

### 2.1. Animals

Heying black chicken was jointly developed by Yangzhou University and Jiangsu Sandali Animal Husbandry Development Co., Ltd., Changzhou, Jiangsu. Through several generations of continuous breeding and cultivation, the production performance has become stable. As a high-quality black-feathered chicken, Heying black chicken has the characteristics of low fat, high protein, rich nutrition and delicious meat. The experimental birds for this study were from the seventh generation of the Heying black chicken B line, which was named after a local chicken breed. A total of 176 chickens were raised under the same conditions in Jiangsu Heying Animal Husbandry Co., Ltd., Changzhou, Jiangsu. They were raised and then slaughtered at the age of 16 weeks. The living environment and nutrition level of chickens were the same. The 176 Heying black chickens (133 hens and 43 roosters) were randomly selected, and the body weight at 0, 2, 4, 8, 10 and 16 weeks of age were recorded. In total, 2.0 mL of blood were collected from the wing vein, anticoagulated with sodium citrate, and stored in a freezer at −20 °C. The 176 Heying black chickens were slaughtered and measured at 16 weeks, and 14 carcass traits including slaughter weights, live weights, semi-eviscerated weights (on the basis of slaughter weights, the weights after removing internal organs) and eviscerated weights (on the basis of semi-eviscerated weight, the weight after removing heart, liver and fat), breast muscle weights (bilateral pectoralis major muscles), leg muscle weights (all muscles from both thighs), head weights, foot weights (foot and shanks), heart weights, liver weights, spleen weights, abdominal fat weights, wing weights and stomach weights (muscle stomach and glandular stomach) were recorded.

For chicks in the age range of 1–3 days, the ambient temperature must be meticulously maintained within the interval of 33–35 °C. As the chicks reach 4–7 days of age, the temperature can be gradually decreased to 32–34 °C. During the second week of their growth, an optimal temperature range of 28–30 °C was sustained. In the third week, the temperature was regulated to fall between 25 and 28 °C. By the fourth week, the temperature was adjusted to 22–25 °C. From the fifth week until the time of slaughter, the temperature needed to be stabilized at 20–21 °C to ensure the proper growth and development of the chickens. For the experimental conditions concerning humidity, we maintained a relative humidity level of 65% to 70% for the chickens aged between 1 and 7 days. The humidity can be adjusted to 60–65% at 8–10 days of age. The relative humidity during the period of 11 to 28 days of age was generally maintained at 55–60%. After 28 days of age, the humidity was usually stabilized at around 55%. The chicken coop has a light exposure time of 12 h and a dark exposure time of 12 h. The chickens in the farm were provided with clean, sterile water via a fully automatic water dispenser, allowing them free access to drinking water at all times. The diet was supplemented with feed manually, and the feed was added three times a day. The feed formula consists of the following components: 3.2% wheat bran, 62% corn, 31% soybean meal, 1.3% calcium hydrogen phosphate, 1.2% stone powder, 1% additives and 0.3% salt.

### 2.2. Genomic DNA Extraction

Genomic DNA was extracted from blood by the phenol/chloroform method. An ultraviolet spectrophotometer was used to determine the concentration and purity of DNA. The DNA was stored at −20 °C for PCR amplification at a later date.

### 2.3. Primer Design

Based on the chicken *FTO* gene sequence published in GenBank, seven pairs of primers (P1–P7) were designed using Primer-BLAST (https://www.ncbi.nlm.nih.gov/tools/primer-blast/, accessed on 5 October 2022) to amplify the exons of the *FTO* gene. The primers were synthesized by Sangon Biotech (Shanghai) Co., Ltd., Shanghai, China, and their sequences are presented in Table 1.

### 2.4. PCR Amplification and Sequencing

The reaction mixtures were added in a 200 μL PCR tube plate for pre-denaturation at 94 °C for 3 min. The following PCR thermal profile was used: denaturation at 94 °C for 15 s, primer annealing at 58 °C for 15 s, DNA synthesis at 72 °C for 30 s, 30 cycles and extension at 72 °C for 5 min. PCR products were sent to Sangon Biotech (Shanghai) Co., Ltd., Shanghai, China, for sequencing.

### 2.5. Analysis Software

DNAMAN 5.2 and Chromas 1.62 were used to visualize the sequencing peak map and detect polymorphic sites through sequence alignment.

### 2.6. Statistical Model and Analysis

1. The data were statistically analyzed using the general linear model (GLM) and Multiple Comparisons (version SPSS 25). The model was Yijk = μ + Gj + Sk + eijk [23], where Yijk was the individual phenotypic record, μ was the population mean, Gj was the effect of genotype, Sk was the sex effect, and eijk was the random error. Differences among genotypes were indicated by superscript letters: different uppercase letters represented highly significant differences (*p* < 0.01), different lowercase letters represented significant differences (*p* < 0.05), and the same letters indicated no significant difference (*p* > 0.05)
2. Polymorphism information content (*PIC*) was calculated using the formula
  $$PIC=1-\left(\sum_{i=1}^{n}P_{i}^{2}\right)-\sum_{i=1}^{n-1}\sum_{j=1}^{n}{2P}_{i}^{2}P_{j}^{2}$$
  where *n* is the number of alleles; *P_i_* and *P_j_* are the frequencies of the *i*th and *j*th alleles, respectively.
3. Effective number of alleles (*Ne*) was calculated using the formula
  $$Ne=1/\sum_{i=1}^{n}P_{i}^{2}$$
4. Average heterozygosity was calculated using the formula
  $$H=1-\sum_{i=1}^{n}P_{i}^{2}$$

### 2.7. Spatiotemporal Expression Differences and Tissue Expression Profiles

Leg and pectoral tissues were collected during the embryonic stage (d12, d14, d16, d18, and d21) and growth period (W2, W4, W8, W10, and W16), and heart, liver, spleen, lung, kidney, fat, breast muscles and leg muscles were collected at the 1st and 16th weeks of life. RNA extraction was performed using the VeZol-Pure Total RNA Isolation Kit (Vazyme, Nanjing, China), followed by reverse transcription with HiScript IV RT SuperMix for qPCR (including gDNA wiper, Vazyme, Nanjing, China) and subsequent real-time fluorescence quantification employing ChamQ Universal SYBR qPCR Master Mix (Vazyme, Nanjing, China). The primer sequences used were as follows: *FTO* (forward: TTCACCAAGGCGACCTCTAC; reverse: GCTGAACCGAGGTGAAAAGC) and β-actin (forward: CAGCCATCTTTCTTGGGTAT; reverse: CTGTGATCTCCTTCTGCATCC). The following PCR protocol was applied: initial denaturation (1 min at 95 °C), followed by a three-step amplification program (20 s at 95 °C, 20 s at 60 °C, 20 s at 72 °C) that was repeated 42 times. The 2−ΔΔCt method was used to analyze the real-time PCR data relative to the average value of control. The quantitative real-time PCR (RT-qPCR) results for all genes were statistically tested using Student’s *t*-test.

## 3. Results

### 3.1. PCR Amplification Results

**Figure 1:**
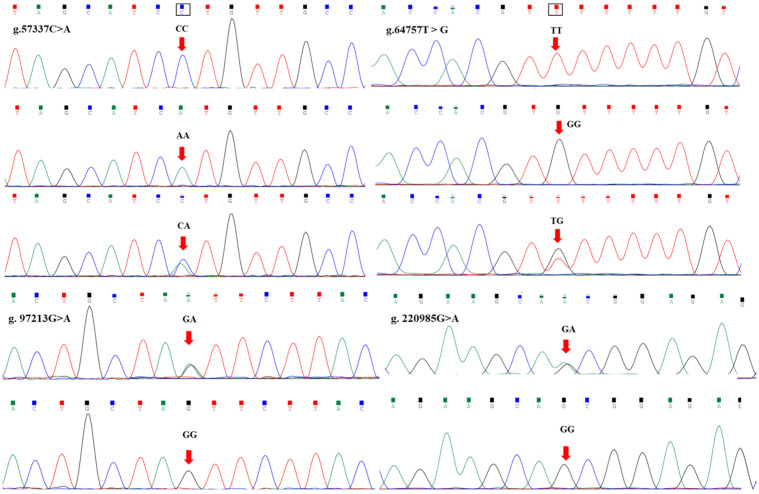
Sequence analysis of mutations in exon regions of the *FTO* gene.

Four SNPs g.57337C>A, g.64757T>G, g.97213G>A, and g.220985G>A were detected in the exon regions of the *FTO* gene using DNA sequencing (Figure 1). The g.57337C>A SNP was in exon 5, and forms *CC*, *AA* and *CA*. The g.64757T>G mutation was in exon 7, and forms *GG*, *TT* and *TG*. The g.97213G>A mutation was in exon 8, and forms *GG* and *GA*. The g.220985G>A mutation was in exon 9, and forms *GG* and *GA*.

### 3.2. Genotyping and Allele Frequency Analysis of the FTO Gene

Polymorphism and genetic diversity analysis of four SNP loci are as shown in Table 2; the gene frequencies of alleles A and C are 0.236 and 0.764. The gene frequencies of alleles T and G were 0.261 and 0.739, respectively. The gene frequencies of alleles G and A were 0.952 and 0.048, respectively. The gene frequencies of alleles G and A were 0.949 and 0.051, respectively. The PICs of alleles g.97213G>A and g.220985G>A were less than 0.25, indicating that they were lowly polymorphic. g.57337C>A and g.64757T>G were moderately polymorphic with PIC values greater than 0.25.

### 3.3. Association Analysis Between the FTO Gene Polymorphisms and Chicken Carcass Traits

#### 3.3.1. Association Analysis of g.57337C>A SNP and Carcass Traits of Heying Black Chicken

SPSS 25.0 was used to analyze the association between the g.57337C>A site of the *FTO* gene and the carcass traits of the Heying black chicken. The analysis showed that the *CA* and *AA* genotype individuals were significantly higher than the *CC* genotype individuals in live weights, head weights, breast muscle weights and leg muscle weights (*p* < 0.05). The *AA* genotype individuals were significantly higher than the *CA* genotype individuals in live weights (*p* < 0.05). The heart weights of *CA* and *AA* genotype individuals were significantly higher than that of *CC* genotype individuals (*p* < 0.01) (Table 3).

#### 3.3.2. Association Analysis of g.64757T>G SNP and Carcass Traits of Heying Black Chicken

The results showed that the *TT* genotype individuals were significantly higher than the *TG* genotype individuals in live weights, eviscerated weights and semi-eviscerated weights (*p* < 0.05). The slaughter weights and wing weights of *TT* and *GG* genotype individuals were significantly higher than those of *TG* genotype individuals (*p* < 0.05) (Table 4).

#### 3.3.3. Association Analysis Between g.97213G>A SNP and Carcass Traits of Heying Black Chicken

The results showed that *GA* genotype individuals were significantly higher than *GG* genotype individuals in slaughter weights (*p* < 0.01), and *GA* genotype individuals were significantly higher than *GG* genotype individuals in live weights, eviscerated weights, semi-eviscerated weights, leg muscle weights and wing weights (*p* < 0.05) (Table 5).

#### 3.3.4. Association Analysis Between g.220985G>A SNP and Carcass Traits of Heying Black Chicken

The *GG* genotype individuals were significantly higher than the *GA* genotype individuals in slaughter weights and live weights (*p* < 0.05) (Table 6). Although *GG* genotype in other tissues was higher than *GA* genotype, it was not significant.

### 3.4. Association Analysis of the FTO Gene Polymorphism and Growth Traits of Heying Black Chicken

#### 3.4.1. Association Analysis of g.57337C>A SNP and Growth Traits of Heying Black Chicken

The association analysis between the *FTO* gene mutation and the growth traits of Heying black chicken showed that the body weights of individuals with the *CA* genotype at 8 weeks was significantly higher than that of individuals with the *CC* genotype (*p* < 0.05), Individuals with the *AA* genotype had body weights between that of individuals with the other two genotypes. The body weights of individuals with *AA* and *CA* genotypes at 10 weeks and 16 weeks were significantly higher than that of individuals with the *CC* genotype (*p* < 0.05) (Table 7).

#### 3.4.2. Association Analysis Between g.64757T>G SNP and Growth Traits of Heying Black Chicken

The body weight of *TT* genotype individuals at 16 weeks was significantly higher than that of *TG* genotype individuals (*p* < 0.05). Body weight of individuals with the *GG* genotype was between that of individuals with the other two genotypes (Table 8).

#### 3.4.3. Association Analysis Between g.97213G>A SNP and Growth Traits of Heying Black Chicken

The weight of *GA* genotype individuals at 8, 10 and 16 weeks of age was significantly higher than that of *GG* genotype individuals (*p* < 0.05) (Table 9). Although *GG* genotype in other weeks was heavier than *GA* genotype, it was not significant.

#### 3.4.4. Association Analysis of g.2220985G>A SNP and Growth Traits of Heying Black Chicken

The weight of individuals with *GG* genotype at the age of 8 weeks, 10 weeks and 16 weeks was significantly higher than that of individuals with *GA* genotype (*p* < 0.05) (Table 10). Although *GG* genotype in other weeks was higher than *GA* genotype, it was not significant.

### 3.5. Spatiotemporal Expression Differences and Tissue Expression Profiles

**Figure 2:**
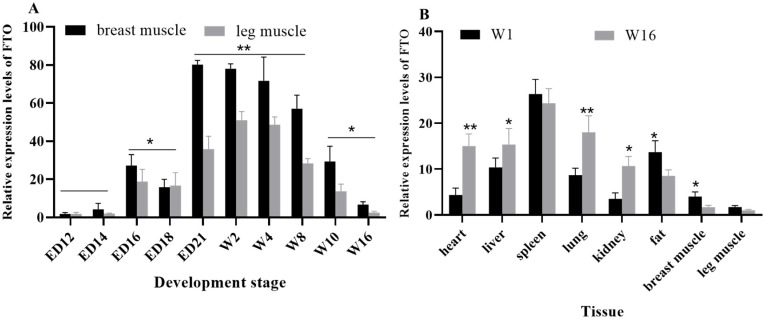
The *FTO* gene mRNA spatiotemporal expression and tissue expression. (**A**) The expression levels of the *FTO* in chicken leg muscle and breast muscle at different stages. (**B**) The expression levels of the *FTO* gene in different tissues of chicken W1 and W16 stages. * *p* < 0.05 and ** *p* < 0.01.

The quantification of the *FTO* gene revealed that expression patterns in the breast muscle and leg muscle followed a similar temporal trajectory (Figure 2A). Notably, the *FTO* gene mRNA expression reached significant levels during the late embryonic and late growth stages (*p* < 0.05), and it was highly significant in the early growth stage (*p* < 0.01). Additionally, *FTO* gene mRNA expression varied across different tissues in an age-dependent manner (Figure 2B). Specifically, at 16 weeks of age, the heart and lungs exhibited significantly higher the *FTO* gene mRNA expression compared to their levels at 1 week of age (*p* < 0.01), while the liver and kidneys showed a significant increase (*p* < 0.05). Conversely, fat tissue and breast muscle displayed significantly higher *FTO* gene mRNA expression at 1 week of age than at 16 weeks (*p* < 0.05).

## 4. Discussion

At present, the research on the *FTO* gene polymorphism is mainly focused on humans. Most studies focus on the relationship between the *FTO* gene variation and obesity [24], body mass index [25], metabolic disease and tumor [26] in different regions and ethnic groups. The *FTO* gene is also widely distributed among various tissues of animals. Chung [27] studied the association between the SNP site of the *FTO* gene and the meat quality traits of Korean cattle. The study showed that the mutation of g.125550A>T site has a significant association with the meat quality traits; Fan et al. [28] found a highly significant association between the pig’s total intramuscular fat content and the c.594C>G mutation of the *FTO* gene. Fu et al. [29] detected the *FTO* gene polymorphism in pigs and found that the G allele at A227G seems to have a beneficial effect on fat deposition. This indicates that the *FTO* gene may participate in the mechanism of fat deposition and is one of the main genes affecting meat quality traits. These studies further showed that the mutation site of the *FTO* gene was significantly related to the slaughter and growth traits of livestock, and may be one of the important candidate genes for the economic traits of livestock and poultry. Improvements in growth and carcass traits are the main objectives for chicken breeding.

To date, the association analysis between the *FTO* gene polymorphism and chicken carcass traits and growth traits has not been reported, and whether it can be used for molecular marker-assisted selection of chicken growth and carcass traits remains to be uncovered. Exons, as a part of eukaryotic genes, contain the core information required for protein synthesis. In this study, primers were designed to detect the SNP sites in the exons of the *FTO* gene of Heying black chicken by PCR synthesis and DNA sequencing technology. Four mutation sites were detected, which were g.57337C>A, g.64757T>G, g.97213G>A and g.220985G>A.

The genotypes of the four mutation sites are significantly related to live weights and slaughter weights. Live weight refers to the weight 12 h after feeding before slaughter. Some studies have shown that heavier broilers (>3.3 kg) produce less breast meat (<3.0 and 3.0 to 3.3 kg) [30]. The slaughter weight refers to the weight of a chicken after bloodletting, hair removal, foot removal (below the tarsal bones) and head removal. The ratio of slaughter weights to live weight determines the slaughter rate of poultry. It is well known that skeletal muscle, as the body’s largest organ, accounts for about 40~50% of the total body weight [31]. The semi-eviscerated weights, and leg and breast muscles are closely related to the development of skeletal muscle. This study showed that g.64757T>G and g.97213G>A were significantly associated with the semi-eviscerated weights (on the basis of slaughter weights, the weight after removing internal organs) and eviscerated weights (on the basis of semi clean bore weight, the weight after removing heart, liver, and fat). Polymorphisms g.57337C>A and g.97213G>A 356 were significantly associated with the leg muscle weights and breast muscle weights. In addition, g.57337C>A was significantly associated with head weights, while g.64757T>G and g.97213G>A were significantly associated with wing weights. Interestingly, our research found that in addition to the traits related to skeletal muscle development, the *FTO* gene also has different associations with some internal organs. For example, g.57337C>A is significantly related to liver weights and heart weights, which needs further exploration and research.

Growth traits are important variables to evaluate the profitability of broiler production. Carcass development often parallels changes in growth traits, with most carcass traits showing significant positive associations [32]. This study showed that four mutation sites (g.57337C>A, g.64757T>G, g.97213G>A and g.220985G>A) were significantly associated with body weights at 16 weeks of age. In addition, g.57337C>A, g.97213G>A and g.220985G>A were significantly associated with body weights at the age of 8 weeks and 10 weeks. Wang et al. [33] showed that the polymorphism formed by insertion/deletion (InDel) of the *FTO* gene was significantly related to the growth traits of Tongyang sheep and could be used to in marker-assisted selection for Tongyang sheep. Polymorphisms in the *FTO* gene showed significant associations with body weights of different breeds of rabbits at various ages, reflecting variations in the effects of the *FTO* gene among species [21].

In conclusion, the four SNPs of the *FTO* gene showed significant association with the slaughter traits related to the skeletal muscle development of Heying black chicken, and also showed significant association with the late growth stage (8, 10 and 16 weeks old). The findings suggest that the *FTO* gene may be a candidate gene related to chicken growth and slaughter traits.

It is quite intriguing to observe that the *FTO* gene expression remains relatively low during the early stages of embryonic development, then gradually increases in the later embryonic phases, persisting through the growth stage, before ultimately declining around 8 weeks of age in the later stages of life. However, research has demonstrated that the level of the *FTO* gene mRNA in the skeletal muscle of 8-week-old chickens is actually higher than that observed in 4-week-old chickens [34]. Wang found that the *FTO* gene is highly expressed in the hypothalamus, liver, visceral fat and cerebellum of chickens [35]. This study found that the *FTO* gene is expressed differently in chicken heart, liver, spleen, lung, kidney, fat, breast muscles and leg muscles. The observed differences in the temporal expression pattern of the *FTO* gene between the present study and previous research may be attributed to variations in chicken breeds.

## 5. Conclusions

This study detected a mutation site in exons 5, 7, 8 and 9 with g.57337C>A, g.64757T>g, g.97213G>A and g.220985G>A, which are homologous mutations. The SNPs have varying degrees of impact on carcass traits. The *FTO* gene may be a candidate gene related to chicken growth and slaughter traits and lays a foundation for Heying black chicken assisted breeding.

## Figures and Tables

**Table 1:** Sanger sequencing of PCR products.

Primer	Sequences (5′–3′)	Tm (°C)	Length (bp)
P1	F: GAGAAGGTTTCCACAACTACCACC R: GTGTGGATGTGTGTGTGACGG	59	205
P2	F: TGCAGTCCTTCAAATACATGATGCT R: CCTCTGGTACGTAACAGCTGC	59	205
P3	F: GGCATATCTATACTTGTGTACTACCCC R: CAGTACTCACCATAAGCTCCATTTTTC	59	209
P4	F: TGGGCAATGTGAACTGGCACTC R: GCACCATGCTTCATGAGCTGTTG	59	202
P5	F: TTTCCTGCACTCAAGGTTTCCTCT R: TGCAGTTCTGGACAAGGCAA	59	218
P6	F: ACTGTCCTTATTTGCTTATGGGCT R: TTACGCAGCCTTTGTCGCTC	59	202
P7	F: ACTGCACAAACGTATCCTTGCTGAG R: AGGCTGGAAGGTGACCTGATATCC	59	253

**Table 2:** Polymorphisms at SNPs of the *FTO* gene.

Locus	Genotype	N	Genotypic Frequency	Allele	Allelic Frequency	*χ*2 Value	*p* Value	*H*	*PIC*	*Ne*
g.57337C>A	AA	18	0.102	A	0.236	11.81	0.003	0.360	0.295	1.563
	AC	47	0.267	C	0.764					
	CC	111	0.631							
g.64757T>G	TT	21	0.119	T	0.261	12.29	0.002	0.386	0.312	1.629
	TG	50	0.284	G	0.739					
	GG	105	0.597							
g.97213G>A	GG	159	0.903	G	0.952	0.45	0.798	0.091	0.087	1.101
	GA	17	0.097	A	0.048					
g.220985G>A	GG	158	0.898	G	0.949	0.51	0.744	0.097	0.092	1.107
	GA	18	0.102	A	0.051					

Note: PIC > 0.5 is highly polymorphic; 0.25 < PIC < 0.5 is moderately polymorphic; PIC < 0.25 means lowly polymorphic.

**Table 3:** Association analysis of g.57337C>A SNP and carcass traits of the Heying black chicken population.

Traits	Genotype		
	*CC* (18)	*CA* (47)	*AA* (111)
Slaughter weights (g)	1171.67 ± 170.68 ^b^	1230.11 ± 266.58 ^ab^	1255.39 ± 230.24 ^a^
Live weights (g)	1348.61 ± 188.01 ^b^	1430.15 ± 262.60 ^a^	1447 ± 241.98 ^a^
Eviscerated weights (g)	880.96 ± 131.11	939.04 ± 182.82	942.60 ± 174.96
Semi-eviscerated weights (g)	1064.98 ± 163.20	1127.60 ± 217.13	1139.87 ± 207.96
Breast muscle weights (g)	62.75 ± 10.65 ^b^	71.57 ± 13.88 ^a^	70.70 ± 11.13 ^a^
Leg muscle weights (g)	89.77 ± 13.59 ^b^	98.54 ± 22.70 ^a^	99.33 ± 23.79 ^a^
Head weights (g)	38.86 ± 7.97 ^b^	44.07 ± 12.22 ^a^	44.30 ± 15.43 ^a^
Foot weights (g)	40.51 ± 7.18	43.75 ± 11.28	42.87 ± 10.97
Heart weights (g)	5.96 ± 0.94 ^B^	7.06 ± 2.41 ^A^	6.99 ± 2.33 ^A^
Liver weights (g)	22.12 ± 3.29 ^b^	23.17 ± 5.33 ^ab^	23.74 ± 4.94 ^a^
Spleen weights (g)	2.70 ± 0.68	2.80 ± 0.72	2.77 ± 0.75
Abdominal fat weights (g)	38.49 ± 21.71	29.60 ± 20.73	34.74 ± 22.99
Wing weights (g)	55.22 ± 6.51	56.92 ± 10.27	57.19 ± 10.73
Stomach weights (g)	34.38 ± 5.62	32.40 ± 5.66	31.24 ± 5.87

Note: In the same line, the differences were highly significant with different uppercase letters of shoulder markers (*p* < 0.01), significantly with different lowercase letters of shoulder markers (*p* < 0.05), and the same letter in shoulder marker indicated no significant difference (*p* > 0.05).

**Table 4:** Association analysis of g.64757T>G SNP and carcass traits of the Heying black chicken population.

Traits	Genotype		
	*GG* (105)	*TG* (50)	*TT* (21)
Slaughter weights (g)	1253.79 ± 236.98 ^a^	1191.30 ± 250.25 ^b^	1302.14 ± 183.56 ^a^
Live weights (g)	1447.30 ± 256.28 ^ab^	1387.70 ± 239.45 ^b^	1487.00 ± 182.61 ^a^
Eviscerated weights (g)	941.44 ± 181.32 ^ab^	906.17 ± 170.28 ^b^	984.42 ± 139.18 ^a^
Semi-eviscerated weights (g)	1138.99 ± 213.88 ^ab^	1091.48 ± 208.60 ^b^	1182.19 ± 165.66 ^a^
Breast muscle weights (g)	69.88 ± 12.35	68.52 ± 12.21	73.40 ± 11.81
Leg muscle weights (g)	98.68 ± 22.57	95.77 ± 24.20	103.25 ± 21.12
Head weights (g)	43.73 ± 13.79	44.00 ± 15.16	43.52 ± 13.44
Foot weights (g)	43.83 ± 11.66	41.05 ± 9.55	44.15 ± 8.54
Heart weights (g)	6.90 ± 2.34	6.79 ± 2.14	7.37 ± 2.44
Liver weights (g)	23.45 ± 5.29	22.95 ± 4.71	24.74 ± 3.09
Spleen weights (g)	2.78 ± 0.80	2.65 ± 0.63	3.00 ± 0.61
Abdominal fat weights (g)	34.83 ± 22.95	30.19 ± 19.76	37.94 ± 24.96
Wing weights (g)	57.73 ± 11.50 ^a^	54.76 ± 10.28 ^b^	59.17 ± 8.80 ^a^
Stomach weights (g)	32.21 ± 5.55	30.74 ± 5.83	32.41 ± 7.09

**Table 5:** Association analysis of g.97213G>A SNP and carcass traits of the Heying black chicken population.

Traits	Genotype	
	*GG* (160)	*GA* (16)
Slaughter weights (g)	1232.05 ± 227.43 ^B^	1339.06 ± 249.43 ^A^
Live weights (g)	1424.88 ± 233.30 ^b^	1537.75 ± 258.91 ^a^
Eviscerated weights (g)	929.13 ± 165.55 ^b^	1015.52 ± 201.60 ^a^
Semi-eviscerated weights (g)	1122.34 ± 198.34 ^b^	1223.16 ± 231.42 ^a^
Breast muscle weights (g)	69.84 ± 11.99	71.25 ± 13.21
Leg muscle weights (g)	97.51 ± 22.47 ^b^	106.24 ± 21.89 ^a^
Head weights (g)	43.47 ± 13.93	46.21 ± 15.00
Foot weights (g)	42.57 ± 10.35	47.01 ± 11.93
Heart weights (g)	6.84 ± 2.19	7.56 ± 2.79
Liver weights (g)	23.27 ± 4.70	25.43 ± 5.58
Spleen weights (g)	2.76 ± 0.74	2.93 ± 0.72
Abdominal fat weights (g)	33.66 ± 22.35	38.14 ± 23.65
Wing weights (g)	56.38 ± 10.17 ^b^	64.11 ± 14.01 ^a^
Stomach weights (g)	31.79 ± 5.86	32.24 ± 5.33

**Table 6:** Association analysis between g.220985G>A SNP and carcass traits of the Heying black chicken population.

Traits	Genotype	
	*GA* (23)	*GG* (153)
Slaughter weights (g)	1160.43 ± 261.05 ^b^	1253.02 ± 229.92 ^a^
Live weights (g)	1376.26 ± 224.17 ^b^	1442.77 ± 245.89 ^a^
Eviscerated weights (g)	892.24 ± 157.07	942.29 ± 175.44
Semi-eviscerated weights (g)	1074.85 ± 200.41	1138.03 ± 207.42
Breast muscle weights (g)	68.57 ± 12.34	70.10 ± 12.23
Leg muscle weights (g)	94.65 ± 16.74	98.80 ± 23.55
Head weights (g)	41.04 ± 12.78	44.08 ± 14.20
Foot weights (g)	40.20 ± 9.58	43.42 ± 10.88
Heart weights (g)	6.34 ± 2.01	7.01 ± 2.31
Liver weights (g)	22.27 ± 4.69	23.64 ± 4.95
Spleen weights (g)	2.68 ± 0.59	2.78 ± 0.76
Abdominal fat weights (g)	30.32 ± 21.94	34.55 ± 22.40
Wing weights (g)	55.73 ± 11.93	57.26 ± 10.73
Stomach weights (g)	30.80 ± 7.06	32.00 ± 5.64

**Table 7:** Association analysis of g.57337C>A SNP and growth traits of the Heying black chicken population.

Weight	Genotype		
	*CC* (17)	*CA* (45)	*AA* (114)
0 week (g)	26.14 ± 2.23	24.39 ± 1.81	25.24 ± 2.12
2 weeks (g)	110.49 ± 15.88	109.56 ± 15.86	109.37 ± 16.11
4 weeks (g)	236.68 ± 35.88	242.01 ± 38.53	235.34 ± 41.03
6 weeks (g)	407.77 ± 50.36	428.80 ± 59.01	416.84 ± 69.54
8 weeks (g)	621.54 ± 66.31 ^b^	687.45 ± 101.79 ^a^	665.46 ± 109.55 ^ab^
10 weeks (g)	836.01 ± 85.83 ^b^	921.08 ± 135.18 ^a^	901.32 ± 139.28 ^a^
16 weeks (g)	1348.61 ± 188.01 ^b^	1430.15 ± 262.60 ^a^	1447 ± 241.98 ^a^

**Table 8:** Association analysis between g.64757T>G SNP and growth traits of Heying black chicken population.

Weight	Genotype		
	*GG* (106)	*TG* (47)	*TT* (23)
0 week (g)	25.14 ± 2.15	25.07 ± 2.10	24.98 ± 2.24
2 weeks (g)	107.47 ± 18.73	112.59 ± 15.62	109.90 ± 19.18
4 weeks (g)	236.03 ± 38.36	240.18 ± 39.22	237.80 ± 48.29
6 weeks (g)	418.39 ± 62.39	423.02 ± 65.45	425.95 ± 82.78
8 weeks (g)	671.33 ± 104.62	665.96 ± 108.58	672.18 ± 107.91
10 weeks (g)	899.77 ± 137.56	911.09 ± 142.86	906.77 ± 125.44
16 weeks (g)	1447.30 ± 256.28 ^ab^	1387.70 ± 239.45 ^b^	1487.00 ± 182.61 ^a^

**Table 9:** Association analysis between g.97213G>A SNP and growth traits of the Heying black chicken population.

Weight	Genotype	
	*GG* (159)	*GA* (17)
0 week (g)	25.14 ± 2.09	24.61 ± 2.29
2 weeks (g)	109.38 ± 16.14	116.11 ± 12.85
4 weeks (g)	236.15 ± 40.77	252.25 ± 25.70
6 weeks (g)	415.92 ± 65.60	453.46 ± 56.75
8 weeks (g)	661.68 ± 102.45 ^b^	732.12 ± 111.30 ^a^
10 weeks (g)	893.42 ± 130.42 ^b^	976.14 ± 170.47 ^a^
16 weeks (g)	1424.88 ± 233.30 ^b^	1537.75 ± 258.91 ^a^

**Table 10:** Association analysis between g.2220985G>A SNP and growth traits of the Heying black chicken population.

Weight	Genotype	
	*GG* (153)	*GA* (23)
0 week (g)	25.31 ± 2.07	23.68 ± 1.83
2 weeks (g)	110.41 ± 16.16	108.19 ± 14.53
4 weeks (g)	239.91 ± 40.36	225.46 ± 32.68
6 weeks (g)	423.24 ± 67.39	398.34 ± 45.99
8 weeks (g)	674.60 ± 107.66 ^a^	631.47 ± 77.56 ^b^
10 weeks (g)	910.29 ± 139.14 ^a^	844.80 ± 103.84 ^b^
16 weeks (g)	1442.77 ± 245.89 ^a^	1376.26 ± 224.17 ^b^

## Data Availability

The data that support the findings of this study are available from the corresponding author upon reasonable request.
